# Disruption of *Var2csa* Gene Impairs Placental Malaria Associated Adhesion Phenotype

**DOI:** 10.1371/journal.pone.0000910

**Published:** 2007-09-19

**Authors:** Nicola K. Viebig, Emily Levin, Sébastien Dechavanne, Stephen J. Rogerson, Jürg Gysin, Joseph D. Smith, Artur Scherf, Benoit Gamain

**Affiliations:** 1 Unité de Biologie des Interactions Hôte-Parasite, Institut Pasteur and CNRS, Paris, France; 2 Seattle Biomedical Research Institute, Seattle, Washington, United States of America; 3 Unité de Parasitologie Expérimentale, Université de la Méditerranée, Marseille, France; 4 Department of Medicine, University of Melbourne, Royal Melbourne Hospital, Parkville, Victoria, Australia; Columbia University, United States of America

## Abstract

Infection with *Plasmodium falciparum* during pregnancy is one of the major causes of malaria related morbidity and mortality in newborn and mothers. The complications of pregnancy-associated malaria result mainly from massive adhesion of *Plasmodium falciparum*-infected erythrocytes (IE) to chondroitin sulfate A (CSA) present in the placental intervillous blood spaces. Var2CSA, a member of the *P. falciparum* erythrocyte membrane protein 1 (PfEMP1) family is the predominant parasite ligand mediating CSA binding. However, experimental evidence suggests that other host receptors, such as hyaluronic acid (HA) and the neonatal Fc receptor, may also support placental binding. Here we used parasites in which *var2csa* was genetically disrupted to evaluate the contribution of these receptors to placental sequestration and to identify additional adhesion receptors that may be involved in pregnancy-associated malaria. By comparison to the wild-type parasites, the FCR3Δvar2csa mutants could not be selected for HA adhesion, indicating that *var2csa* is not only essential for IE cytoadhesion to the placental receptor CSA, but also to HA. However, further studies using different pure sources of HA revealed that the previously observed binding results from CSA contamination in the bovine vitreous humor HA preparation. To identify CSA-independent placental interactions, FCR3Δvar2csa mutant parasites were selected for adhesion to the human placental trophoblastic BeWo cell line. BeWo selected parasites revealed a multi-phenotypic adhesion population expressing multiple *var* genes. However, these parasites did not cytoadhere specifically to the syncytiotrophoblast lining of placental cryosections and were not recognized by sera from malaria-exposed women in a parity dependent manner, indicating that the surface molecules present on the surface of the BeWo selected population are not specifically expressed during the course of pregnancy-associated malaria. Taken together, these results demonstrate that the placental malaria associated phenotype can not be restored in FCR3Δvar2csa mutant parasites and highlight the key role of var2CSA in pregnancy malaria pathogenesis and for vaccine development.

## Introduction

*Plasmodium falciparum* causes the most severe form of human malaria, with over two million deaths per year. At particular risk of developing severe, life-threatening malaria-associated complications are children and women during their first pregnancy [1]. Whereas adults in high transmission regions usually develop protective clinical immunity to malaria, primigravid women are highly susceptible to a placental form of infection [2]. Complications of pregnancy-associated malaria (PAM) result mainly from massive sequestration of *Plasmodium falciparum*-infected erythrocytes (IE) in the placental intervillous blood spaces [3]. Placental sequestration impacts both mother and fetus, contributing to premature delivery, intrauterine growth retardation, stillbirth, maternal anaemia, and increased neonatal and maternal mortality [4]. Whereas sequestration in the peripheral microvasculature is associated with IE that bind CD36 and variably to other host receptors, chondroitin sulfate A (CSA) expressed by placental syncytiotrophoblasts has been described as a common receptor involved in IE placental sequestration [3]. With successive pregnancies, women develop protective antibody responses that block CSA binding and recognize geographically diverse placental isolates [5], [6], suggesting that a vaccine against PAM is feasible. To design a vaccine to protect pregnant women and their fetuses, it is therefore crucial to define the range of host receptors and parasite ligands involved in placental sequestration.

Cytoadhesion is mediated through the *P. falciparum* erythrocyte membrane protein-1 (PfEMP1), encoded by members of the *var* multi gene family [7]–[9]. Gene disruption has been used to show that var2CSA is the primary PfEMP1 protein mediating CSA-binding and the only CSA-binding protein that displays a placental antigenic phenotype [10], [11]. However, it is still controversial if CSA is the only placental receptor involved during PAM. Therefore if additional host receptors are involved, the corresponding parasite ligands need to be characterized in order to develop efficient vaccines. Experimental evidence suggests that IE in the placenta interact with neonatal Fc receptors via surface bound non-immune IgG [12] and cytoadhere to hyaluronic acid (HA) [13], [14]. Therefore, FCR3Δvar2csa mutant parasites are not only a useful tool to evaluate if additional PfEMP1 besides var2CSA have a role in placental IE cytoadhesion, but could also identify additional host receptors on the syncytiotrophoblasts or in the placental intervillous space.

In this study, we used FCR3Δvar2csa mutant parasites to investigate if *var2csa* is essential for HA cytoadhesion and if the parasite genome encodes for other parasite ligands that mediate binding to this receptor. In addition, we used the human placental-derived trophoblastic BeWo cell line [15]–[17] to identify other putative unknown receptors present on the surface of syncytiotrophoblasts that could play a role in placental sequestration. Using these approaches, we were unable to define new parasite adhesion ligands beyond var2CSA that were recognized by sera of malaria-exposed women in a parity dependent manner. Our results strongly support the concept that the massive accumulation of IE in the placenta is predominantly mediated through CSA specific cytoadhesion and that var2CSA is the key virulence factor involved in the pathogenesis of PAM.

## Results

### Var2csa is essential for IE cytoadhesion to purified hyaluronic acid preparations

**Figure 1:**
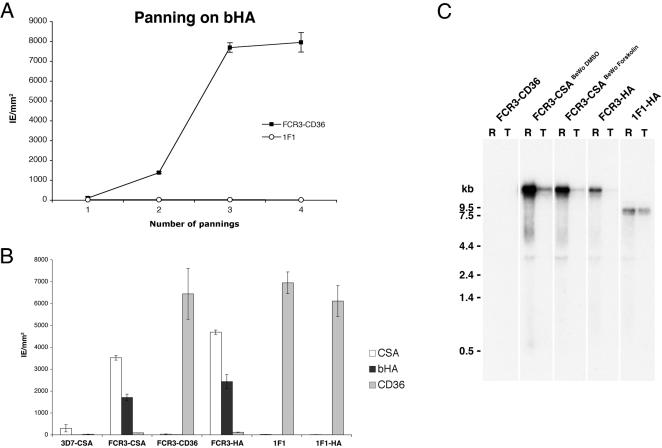
Var2csa is essential for IE cytoadhesion to hyaluronic acid. A. Adhesion profiles of *P. falciparum* infected-erythrocytes during selection on bHA. Trophozoite-stage *P. falciparum* FCR3-CD36 and FCR3Δvar2csa 1F1 were subjected to four rounds of selection on plastic Petri dishes coated with bHA. Mean numbers (±SD) of IE adhering per mm^2^ for four different fields are shown. B. FCR3-HA IE cytoadhere to CSA and HA coated to plastic. Erythrocytes infected with *P. falciparum* FCR3-CSA, 3D7-CSA, FCR3-CD36, FCR3Δvar2csa 1F1 and the parasites selected on HA FCR3-HA and 1F1-HA were analyzed for cytoadhesion to CSA (white bars), bHA (dark gray bars) or CD36 (light gray bars). Data are the mean number (±SEM) of IE per mm^2^ adhering to receptor-coated plastic Petri dishes, as determined in two independent assays in duplicate spots. C. Northern blot analysis of total RNA isolated from ring (R) and trophozoite stage parasites (T) FCR3-CD36, FCR3-CSA ^BeWo DMSO^, FCR3-CSA ^BeWo Forskolin^, FCR3-HA and the HA-selected FCR3Δvar2csa clone 1F1-HA. The membrane was hybridized with a probe specific for *var2csa* DBL1-X. The 1F1-HA gene disruption parasite constitutively expresses a truncated, non-functional *var2csa* transcript [10], which explains the lower molecular weight product in these lanes.

*Var2csa* was previously reported to be transcriptionally upregulated in both CSA [18], [19] and HA binding parasites [20]. To evaluate if other PfEMP1 besides var2CSA could mediate IE cytoadhesion to HA, we tested the capability of the FCR3Δvar2csa mutant clone 1F1 to gain a HA binding phenotype upon selection on bovine HA (bHA) immobilized to plastic Petri dishes. Whereas FCR3-CD36 wild type parasites, displaying a CD36 binding phenotype before selection, gained binding to bHA after only four rounds of selection, no specific enrichment was observed for the 1F1 FCR3Δvar2csa mutants (Fig. 1A).

The HA-selected IE adhesion phenotype was further examined on bHA, CSA and CD36 coated to plastic Petri dishes (Fig. 1B). While wild type FCR3 IE selected on bHA (FCR3-HA) or with the CSA binding phenotype (FCR3-CSA) bound to bHA and in higher numbers to CSA, 1F1 FCR3Δvar2csa mutant clone IE selected on bHA (1F1-HA) maintained their CD36 binding phenotype and did not acquire any binding to CSA and bHA (Fig. 1B). In comparison to the FCR3-CSA IE, 3D7-CSA IE revealed a very low binding to CSA and no binding to bHA. No cytoadhesion to BSA or CSC was observed for any of the IE (data not shown). As expected from previous studies [18]–[20], FCR3-CSA and FCR3-HA parasites transcribed a full-length *var2csa* transcript, while FCR3Δvar2csa 1F1-HA IE transcribed a truncated non-functional *var2csa* transcript [10] (Fig. 1C). Furthermore, using a probe to the semiconserved *var* exon II, transcripts of around 9 kb were identified in ring-stage RNA of FCR3-CD36 and in the mutant clone 1F1-HA (data not shown). Taken together, these data indicate that *var2csa* is essential for cytoadhesion of the late stage FCR3-IE to both the placental receptor CSA and to bHA and that no other *var* genes in FCR3Δvar2csa mutants were able to compensate for HA binding in the absence of *var2csa*.

### Evaluation of the HA binding specificity

**Figure 2:**
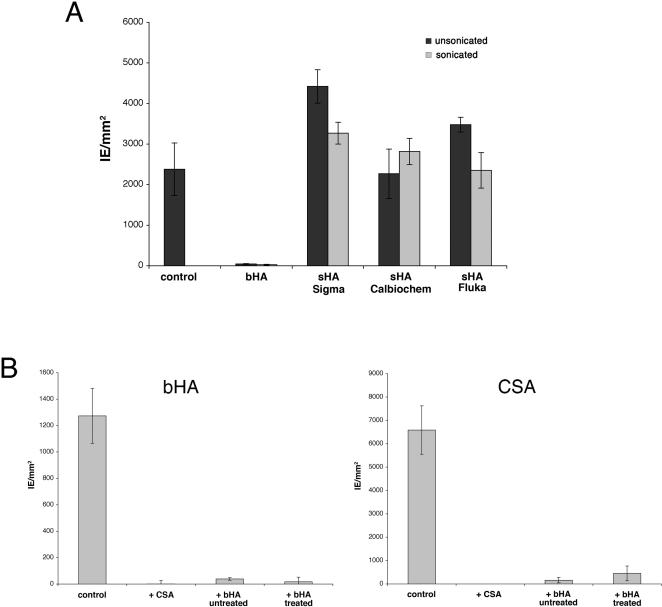
Binding of the FCR3-HA selected parasites to HA and CSA is inhibited by soluble bovine HA and soluble CSA, but not by *Streptococcus* HA. A. Evaluation of the HA binding specificity of the FCR3-CD36 IE selected on HA was determined without (control) or with 200 µg/ml of different HA preparations sonicated or not in the binding medium. HA from bovine vitreous humor (bHA) and different HA from *Streptococcus* (sHA) were tested for their ability to inhibit IE binding to bHA. Data are the mean number (±SEM) of IE per mm^2^ adhering to receptor-coated plastic Petri dishes, as determined in three independent assays in duplicate spots. B. Soluble bHA and CSA cross-inhibit cytoadhesion of FCR3-HA IE to bHA as well as CSA. FCR3-HA IE were pre-incubated with 200 µg/ml soluble CSA as well as untreated or treated bHA prior to the binding assay. The ability to inhibit IE cytoadhesion to bHA (left panel) and CSA (right panel) was examined. Data are the mean number (±SEM) of IE per mm^2^ adhering to receptor-coated plastic Petri dishes, as determined in three (A) or two (B) independent assays in duplicate spots.

Although these results seem to implicate var2CSA as being the parasite ligand for HA binding, several studies have raised doubts on the specificity of HA cytoadhesion as it has been reported that bHA preparations used to select IE contain low to moderate levels of CSA contamination [21]–[23]. To test the HA binding specificity of FCR3-HA IE, binding inhibition assays were performed as described previously [24] using *Streptococcus* HA (sHA) preparations known to be free of CSA from three different commercial sources. Sonication has been shown to increase HA binding inhibition activity [24], so HA preparations were tested plus or minus sonication. Whereas bHA with or without sonication treatment totally abrogated IE cytoadhesion to bHA coated on plastic Petri dishes, the three commercially available sources of sHA used in this study completely failed to inhibit binding to bHA, whether sonicated or not (Fig. 2A). Furthermore, soluble CSA or bHA were able to completely cross-inhibit FCR3-HA cytoadhesion to bHA as well as to CSA (Fig. 2B). In addition, chondroitinase ABC treatment, but not *Streptomyces hyaluronlyticus* hyaluronidase treatment inhibited IE adhesion to the two receptors, CSA and bHA (data not shown). Therefore, binding of FCR3 IE to bHA is caused by CSA contamination in the HA preparation.

### Selection of var2csa disrupted IE on the placental BeWo cell line and phenotypic analysis

**Figure 3:**
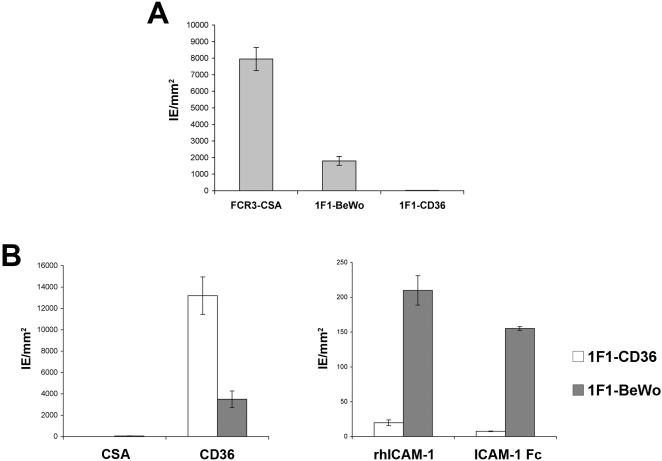
Phenotypic analysis of FCR3Δvar2csa 1F1 IE selected on the human placental derived trophoblastic BeWo cell line. A. Cytoadhesion of IE to BeWo cells was compared for the FCR3Δvar2csa clone 1F1 selected on BeWo cells (1F1-BeWo), the 1F1 control parasites selected on recombinant human CD36 (1F1-CD36) and the wild-type FCR3-CSA parasites. Data are the mean number (±SEM) of IE per mm^2^ adhering to BeWo cells as determined in at least two independent assays in duplicate. B. Erythrocytes infected with *P. falciparum* 1F1-CD36 (white bars) and 1F1-BeWo (gray bars) were analyzed for cytoadhesion to CSA, CD36 (left panel), recombinant human ICAM-1 (rhICAM-1) and ICAM-1 Fc (right panel). Data are the mean number (±SEM) of IE per mm^2^ adhering to receptor-coated plastic Petri dishes as determined in at least two independent assays in duplicate.

Currently, there are no *P. falciparum* animal models for placental sequestration or PAM disease. However, recently, it has been shown that placental isolates adhere strongly to the human placental-derived trophoblastic BeWo cell line and that this is a quick and easy alternative to select IE for the CSA-binding phenotype [15]–[17]. In order to identify other putative unknown receptors present on the surface of syncytiotrophoblasts that could play a role in placental sequestration, we selected the FCR3Δvar2csa mutant clone 1F1 on the BeWo cell line. After six pannings on the BeWo cell line, the 1F1-BeWo selected parasite population bound to the BeWo cells, however, in approximately five-fold lower numbers than the FCR3-CSA wild type parasites (Fig. 3A). In contrast, 1F1 parasites selected to bind CD36 did not bind BeWo cells (Fig. 3A).

BeWo cells are heterogeneous cells [25] expressing numerous potential parasite cytoadhesion receptors including CSA, intercellular adhesion molecule-1 (ICAM-1) [15] and also the neonatal Fc receptor [12], [26]. However, these cells do not express CD36, CD31, E-selectin and vascular cell adhesion molecule-1 (VCAM-1) [15]. To examine the 1F1-BeWo parasites binding phenotype, cytoadhesion experiments to different host receptors coated on plastic Petri dishes were performed. Whereas 1F1-CD36 parasites bound only to CD36, 1F1-BeWo IE adhered in low numbers to two different sources of recombinant human ICAM-1 and to CD36, but did not bind to CSA (Fig. 3B).

**Figure 4:**
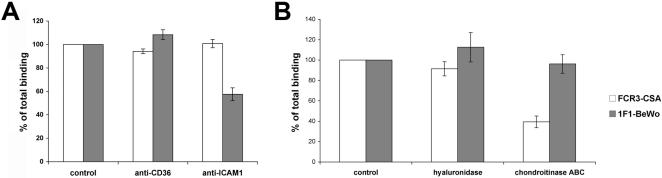
Anti-ICAM-1 antibody inhibits 1F1-BeWo IE binding to BeWo cells. A. and B. Binding specificity of FCR3-CSA (white bars) and 1F1-BeWo IE (gray bars) to BeWo cells was determined using various inhibitors. BeWo cells were either pre-incubated with adhesion blocking anti-ICAM-1 or anti-CD36 antibodies at 5 µg/ml (A) or pre-treated with *Streptomyces hyaluronlyticus* hyaluronidase (25 units/ml) or chondroitinase ABC (0.5 units/ml) for 1 h at 37°C (B). Data are the mean percentage (±SEM) of IE binding compared to the appropriate control as determined in three independent experiments.

To characterize the respective receptors that FCR3-CSA and 1F1-BeWo IE were using to adhere to BeWo cells, binding inhibition assays were performed. While antibodies to ICAM-1 had no effect on FCR3-CSA IE adhesion, they inhibited 1F1-BeWo IE cytoadhesion by 40%, indicating that parts of the population display an ICAM-1 binding phenotype (Fig. 4A). By comparison, chondroitinase ABC treatment of the BeWo cells partially inhibited FCR3-CSA IE cytoadhesion, but 1F1-BeWo IE binding was not altered (Fig. 4B). No cytoadhesion inhibition of either parasite line was observed after either hyaluronidase treatment of the cells or antibodies to CD36 (Fig. 4). These results demonstrate that the 1F1-BeWo IE binding interaction to the BeWo cells is CSA and HA independent.

Binding of non-immune IgG on the IE surface of CSA binding parasites expressing var2CSA such as FCR3-CSA was previously reported to be involved in IE adhesion to the neonatal Fc receptors expressed by the syncytiotrophoblasts [12], [14]. As BeWo cells have been described to express the neonatal Fc receptor [26], we assessed the capacity of FCR3-CSA and 1F1-BeWo IE grown in the presence of human sera to bind to the BeWo cells. Although under our experimental conditions non-immune immunoglobulins can be detected on the surface of BeWo cells and FCR3-CSA IE but not on the surface of the 1F1-BeWo IE (data not shown), pre-incubation of late stage FCR3-CSA and 1F1-BeWo IE with 200 µg/ml protein A, but not with BSA, resulted for both parasite lines in partial and non-specific inhibition of adhesion to the BeWo cells (data not shown). The fact that this partial inhibition was independent of the parasite phenotype and of the presence of immunoglobulins on the IE surface suggests a non-specific inhibition and a minor role for PfEMP1 in this interaction. Taken together, our data shows that selection of FCR3Δvar2csa parasites for cytoadhesion to the placental derived BeWo cell line, results in a CSA-independent, multi-phenotypic parasite population.

### Several var genes are transcribed in 1F1-BeWo parasites

**Figure 5:**
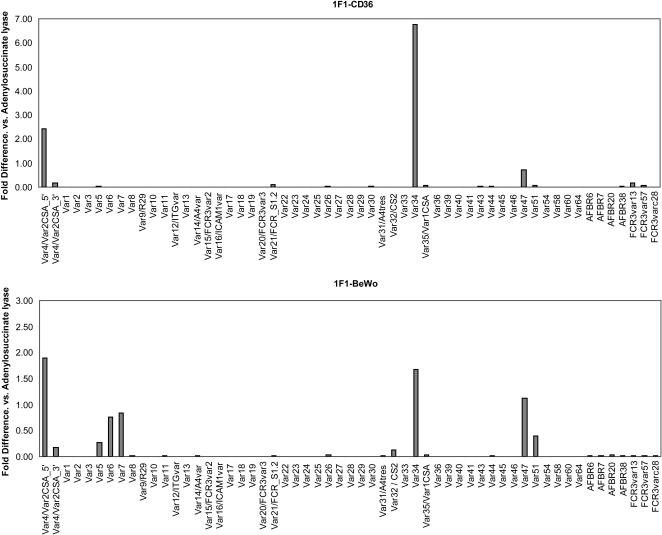
Transcriptional analysis of *var* genes in 1F1-CD36 and 1F1-BeWo parasite lines. Prior to selection, the FCR3Δvar2csa clonal line 1F1 bound CD36. The 1F1 parasite line was reselected on either human recombinant CD36 (top panel) or six times on BeWo cell lines (bottom panel) before *var* transcriptional analysis. Both 1F1-derived parasite lines express a partial, non-functional *var2csa* transcript as a result of the gene integration event [10]. The partial *var2csa* transcript is detected by primers to the 5′ part of the gene, but not to the 3′ end of the gene. The 1F1-CD36 parasite line expresses one dominant *var* gene, *var34*, and a second gene at lower level (*var47*). The *var34* and *var47* transcripts are also present in the 1F1-BeWo parasite line, plus four additional *var* genes (*var5*, *var6*, *var7*, and *var51*).

Cytoadhesion of late stage IE is mainly mediated by PfEMP1 that is encoded by members of the *var* multi gene family [7]–[9]. Recently, the *var* gene repertoire was obtained from the FCR3/CS2/IT4 parasite genotype [27]. To identify the *var* gene(s) predominantly transcribed in the 1F1-CD36 and 1F1-BeWo parasite populations, gene-specific primers were designed to the 56 known *var* genes (materials and methods) and quantitative real-time PCR was performed on RNA extracted from ring stage parasites at 10 h post-invasion (Fig. 5). Prior to *var* transcriptional analysis, 1F1-CD36 IE were reselected on recombinant human CD36 and 1F1-BeWo parasites were analyzed after six rounds of selection on the BeWo cells. As expected, both 1F1-derived parasite lines express a partial, non-functional *var2csa* transcript as a result of the gene disruption event [10]. This transcript was detected by primers to the 5′ part of the gene, but not to the 3′ end of the gene. The 1F1-CD36 parasite line also expresses one dominant *var* gene, *var34*, and a second gene at lower level (*var47*). The *var34* and *var47* transcripts are also present in the 1F1-BeWo parasite population, plus four additional *var* genes (*var5*, *var6*, *var7*, and *var51*) (Fig. 5). These results were confirmed by Northern blot analysis (Fig. S1). The results of this *var* gene transcriptional analysis indicate that the 1F1-BeWo population expressed multiple *var* genes displaying a multi-adhesive phenotype.

### 1F1-BeWo selected parasites do not show characteristics of placental parasites

**Figure 6:**
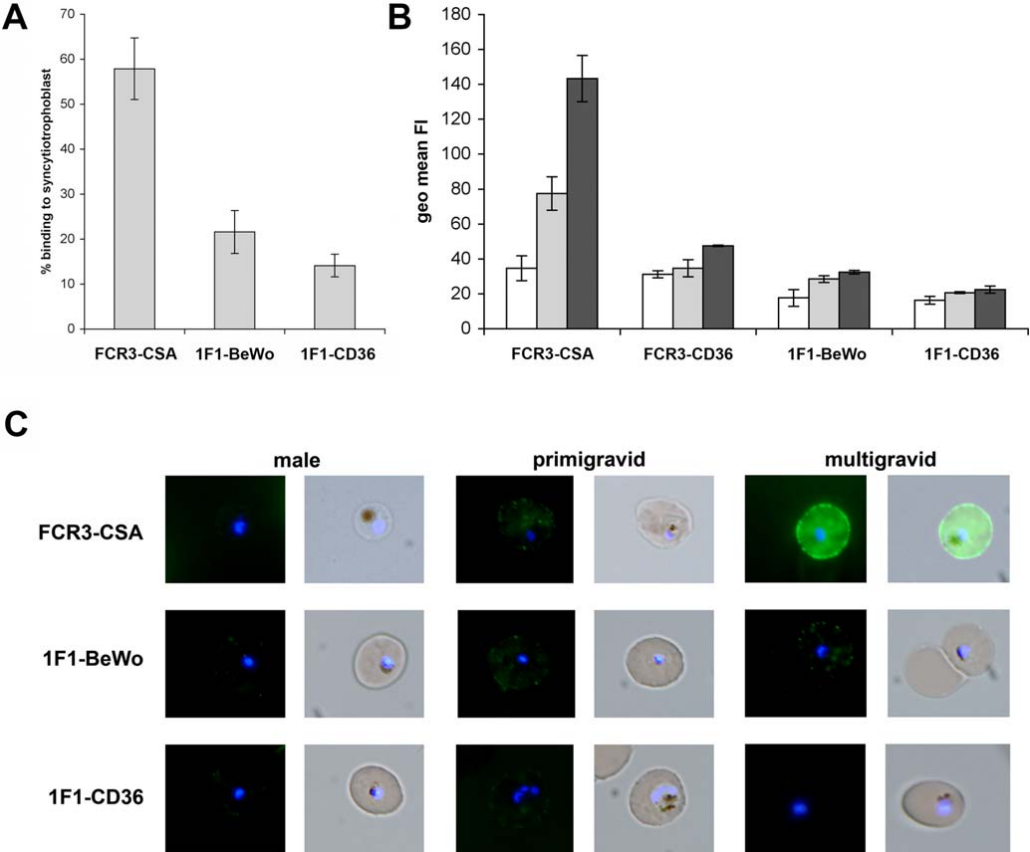
No specific cytoadhesion of 1F1-BeWo IE to placental syncytiotrophoblasts and no parity dependent sera recognition. A. Binding of late stage IE to syncytiotrophoblasts of normal human placenta cryosections under flow conditions at a shear stress of 0.05 Pa. Data shown are the percentage (±SD) of IE bound to the syncytiotrophoblasts versus IE bound in total as determined in three independent experiments on cryosections of two different placentas in duplicate. B and C. Recognition of IE with different binding phenotypes by sera pools of Malawian malaria exposed males (white bars), primigravid (light gray bars) and multigravid women (dark gray bars). Analysis was performed using flow cytometry (B) and fluorescence microscopy (C). Flow cytometry data shown are the mean values of the geometric mean fluorescence intensities (FI) (±SD) for the samples.

The BeWo cell line is considered to be a model that can be used to study interactions between IE and placental syncytiotrophoblasts. To test if the 1F1-BeWo parasite line has the characteristics of a placental parasite population it was investigated for adhesion to normal human placenta cryosections under flow conditions at 0.05 Pa. In this experiment, FCR3-CSA parasites, used as a control, adhered mainly to the syncytiotrophoblast lining. In contrast, 1F1-BeWo and 1F1-CD36 IE showed weaker binding and adhered mostly to the villous tissue and not specifically to the syncytiotrophoblast lining (Fig. 6A, and data not shown). To examine whether 1F1-BeWo selected parasites were recognized in a gender-specific manner they were tested by flow cytometry and live immunofluorescence assay using sera from Malawian male and pregnant women. Using these sera, only FCR3-CSA IE were recognized by sera pools of malaria-exposed women in a parity dependent manner (Fig. 6B and C). In contrast, FCR3-CD36, 1F1-BeWo and 1F1-CD36 were recognized equally well by sera pools of malaria-exposed males, primigravid and multigravid women (Fig. 6B and C). Taken together, the low binding to the syncytiotrophoblast lining and the parity independent surface reactivity suggest that erythrocyte surface molecules expressed by 1F1-BeWo selected population are not specifically expressed during the course of pregnancy-associated malaria.

## Discussion

Cytoadhesion of late stage IE in the placenta is a crucial event in the development of severe malaria complications during pregnancy especially in primigravid women [28]. Although it is generally accepted that a vaccine that would protect against PAM should target the var2CSA molecule, a crucial question remains to be addressed, namely if non-CSA mediated adhesion events could replace CSA adhesion in the placenta. Using our recently described *var2csa* deficient parasite line FCR3Δvar2csa 1F1 [10], we were able to evaluate the role of non-CSA placental adhesion receptors.

In this study, we showed that *var2csa* is linked to the interaction with certain HA preparations bound to plastic and that this phenotype is lost in *var2csa* deficient mutant parasites. However, we failed to prove the specificity of HA in the binding assays, leading us to the conclusion that binding of IE to bHA is mediated by CSA contamination in the HA preparation. These results are in disagreement with several studies reporting HA specific IE cytoadhesion [13], [14], [24], [29], [30], but supported by other studies that raised doubts on the specificity of HA mediated IE cytoadhesion [22], [23], [31]. More recently, Muthusamy et al. demonstrated that HA is not present in the intervillous space of the human placenta [31], indicating that HA is unlikely to be involved in placental sequestration.

In addition to CSA and HA, the binding of non-immune immunoglobulins to the IE surface was reported to be an important ligand for IE adhesion to the placental syncytiotrophoblasts via neonatal Fc receptors [12]. This study implicated a non-*var2csa*-type *var* gene (TM284S2 *var1*) in placental binding. To address whether other parasite ligands can supplant placental binding in the absence of functional var2CSA, we selected 1F1 FCR3Δvar2csa mutant parasites on BeWo cells. We obtained a parasite line that displayed a mixed, but CSA-independent binding phenotype. 1F1-Bewo cells adhered much weaker than FCR3-CSA IE to BeWo cells. This observation supports previous results indicating that var2CSA is the primary ligand for CSA in the FCR3 genotype. The CSA-independent IE adhesion with BeWo cells was investigated for its possible interaction with the neonatal Fc receptor. IE adhesion to the BeWo cells was partially inhibited with protein A. This inhibition was, however, independent of the parasite phenotype and of the presence of immunoglobulins on the IE surface, suggesting a non-specific inhibition and a minor role for PfEMP1 in this interaction. In addition, several studies have concluded that neonatal Fc receptors are not expressed on the syncytiotrophoblasts surface and may therefore not be accessible to IE cytoadhesion [32]–[34]. Taken together, our data show that the neonatal Fc receptor is not a major placental cytoadhesion receptor for FCR3 parasites under the experimental conditions.

To assess the potential role of non-var2CSA PfEMP1 variants in PAM pathogenesis, we characterized the binding and antigenic phenotype of BeWo selected parasites. By comparison to FCR3-CSA IE expressing *var2csa*, 1F1-BeWo IE do not sequester specifically to the syncytiotrophoblast lining of normal human placental cryosections nor are they recognized in a gender-specific manner by endemic sera suggesting they are not expressed during the course of pregnancy-associated malaria.

Similar to our work, Duffy et al. [11] have generated *var2csa*-disruption mutants in a parasite line, termed CS2, which is isogenic to the FCR3 parasite used here. While CS2Δvar2csa were able to weakly recover a CSA-binding phenotype, these minor variants were not recognized by pregnant women sera in a parity dependent manner and therefore are unlikely to have an important role in pregnancy associated malaria. Recently, the *var* gene repertoire was obtained from the FCR3/CS2/IT4 parasite genotype [27]. Interestingly, the *varP* gene tag described to be upregulated in these CS2 *var2csa* deficient mutants is identical to the *var6* gene upregulated in our 1F1-BeWo parasite line. Therefore it is possible that the *var6* protein possesses a very low affinity CSA binding domain. However, none of the *var* genes upregulated in 1F1-BeWo parasites has significant relationship to the TM284S2 *var1* gene [12], which, unlike *var2csa*, is not conserved across *P. falciparum* isolates.

While the new *var* genes upregulated on BeWo-selected IE are unlikely to have a role in PAM pathogenesis, they do provide new information about *var*-associated binding phenotypes. Whereas 1F1-CD36 parasites bound only to CD36, 1F1-BeWo IE adhered in low numbers to human ICAM-1 and to CD36. As the 1F1-CD36 parasites do not bind to ICAM-1 we can exclude that *var34* and *var47* are involved in the observed 1F1-BeWo ICAM-1 binding phenotype. As BeWo cells do not express CD36, the CD36 binding phenotype of 1F1-BeWo likely arose due to a PfEMP1 variant that happened to encode CD36 binding activity function in addition to the host receptor adhesion trait that led to its selection on BeWo cells. This is not surprising because CD36 binding is a common adhesion trait in many different PfEMP1 variants [35]. Because *var34* is found in FCR3 parasites with different phenotypes we suggest that high “on” rates for the *var34 var* gene in FCR3 parasites may explain the frequently observed subpopulation expressing *var34.*

With regard to the development of a vaccine aiming to protect pregnant women and their fetuses from severe disease, our results are encouraging. Using FCR3 parasites and a defined placental binding model, we were unable to confirm a role for HA as well as for non-immune immunoglobulins in IE placental sequestration nor to select for additional parasites with a placental antigenic phenotype, besides var2CSA. Although we cannot exclude that by using another laboratory strain or another selection system we would have observed the same data, our experimental findings strongly point to var2CSA as the main parasite ligand mediating high affinity and high density cytoadhesion to the placenta. Compared to typical PfEMP1 proteins, var2CSA is exceptionally conserved between parasite isolates from different regions of the world [36], [37]. This could help explain how multigravid women in malaria endemic areas develop antibodies that recognize erythrocytes infected with placental and CSA binding parasites in a strain transcendent manner. In conclusion, the analysis of a mutant parasite (*var2csa* KO) has been a valuable tool to reevaluate previously described interactions of IE with the placenta. Our results indicate that vaccine development should focus on the var2CSA molecule.

## Materials and Methods

### Parasite and cell culture

The *P. falciparum* FCR3 and 3D7 strains were cultivated according to standard conditions [38] in O+ human erythrocytes in RPMI 1640 containing L-Glutamine (Invitrogen) supplemented with 5% human serum (PAA Laboratories GmbH), 0.25% Albumax I (Invitrogen), 1× hypoxanthine (c.c.pro) and 20 µg/ml gentamicin (Sigma). FCR3Δvar2csa clone 1F1 [10] was grown in the presence of 2.5 nM WR99210 (Jacobus Pharmaceutical Company). For selection on BeWo cells and for flow cytometry, parasites were grown in RPMI 1640 containing L-Glutamine supplemented with 0.5% Albumax I, 1× hypoxanthine and 20 µg/ml gentamicin. Cultures were synchronized as described previously [39]. To maintain knob-positive parasites, cultures were routinely selected by gelatin flotation using Plasmion (Fresenius Kabi) [40]. The human choriocarcinoma placenta BeWo cell line was cultured as described [15]. Parasites and cells were tested *Mycoplasma* negative by PCR.

### Binding phenotype selection

To select the FCR3 strain and the FCR3Δvar2csa clone 1F1 for specific binding phenotypes, trophozoite-stage parasitized erythrocytes (IE) were purified using Plasmion and selected either on BeWo cells or on plastic Petri dishes coated with bHA or recombinant CD36.

To obtain a CSA-binding phenotype, FCR3-CSA and 3D7-CSA IE used in this study were selected on the trophoblastic BeWo cell line as described recently [15].

Plastic Petri dishes (Falcon #1005) were coated overnight at 4°C with PBS containing 100 µg/ml HA sodium salt from bovine vitreous humor (Sigma) or 10 µg/ml recombinant human CD36-Fc (R&D Systems), washed three times with PBS and blocked with 1% BSA for 1 h at RT, before trophozoite-IE were allowed to adhere. All assays were carried out using RPMI 1640/25 mM Hepes, pH 7.2 (panning buffer). BeWo cells were washed three times with panning buffer, before IE were allowed to adhere. For gelatin enrichment, 1 ml RBC pellet was mixed with 1.4 ml of parasite culture medium and 2.4 ml of Plasmion and incubated for 30 min in a 37°C water bath. The enriched IE were washed twice with panning buffer and were resuspended in 10 ml of panning buffer at a concentration of approximately 5×10^7^ IE/ml. After incubation at 37°C in a 5% CO_2_ incubator for 1 h with gentle agitation every 15 min, non-adherent IE were washed away with panning buffer. Bound IE were detached with the pipette stream and returned to culture. Parasites were grown to a parasitemia of 4–10% before repeating this process three times for selection on bHA, five times for selection on BeWo cells.

### Cytoadhesion assays on immobilized receptors

Cytoadhesion assays on receptors immobilized on plastic petri dishes were carried out as described [41], [42]. Briefly, plastic Petri dishes were coated overnight at 4°C with PBS containing 1 mg/ml CSA sodium salt from bovine trachea (Sigma), 1 mg/ml chondroitin sulfate C sodium salt from shark cartilage (CSC) (Sigma), 100 µg/ml HA sodium salt from bovine vitreous humor (Sigma), 10 µg/ml recombinant human ICAM-1/Fc Chimera (R&D Systems), 10 µg/ml recombinant human ICAM-1 (R&D Systems), 1% BSA or MAb 179 (25 µg/ml) [41]. MAb 179 coated spots were incubated with recombinant CD36 protein containing this epitope tag for 1 h at RT. All spots were blocked with 1% BSA for 1 h at RT before trophozoite-IE (5×10^7^ IE/ml) were allowed to adhere. The average number of adherent IE (±SEM) for four different fields in duplicate spots was determined in two to three independent experiments after fixing with 2% glutaraldehyde in PBS for 2 h at RT and staining the plates with Giemsa. Pictures were taken with a Nikon camera. Lucia software was used to determine the number of bound IE.

### Inhibition assays on immobilized receptors

HA of the different sources was dissolved overnight at 4°C on a rotating wheel at a concentration of 5 mg/ml in water. The solutions were treated by intermittent sonication, in an ice water bath, for 5 min using a Bioruptor (Diagenode) set at maximum output with 30 sec/30 sec intervals. Control HA samples were incubated in an ice water bath for an equivalent time. HA from bovine vitreous humor (Sigma), *Streptococcus sp.* (Calbiochem), *Streptococcus equi* (Fluka) and *Streptococcus zooepidemicus* (Sigma) was tested. CSA was dissolved in PBS at a concentration of 5 mg/ml. 5×10^7^ IE/ml were pre-incubated with 200 µg/ml of the different soluble HA preparations for 30 min at RT and adding the soluble inhibitors to the binding assay. 200 µg/ml CSA was used as a positive control for inhibition of cytoadhesion.

### Inhibition assays on BeWo cells

Specificity of IE binding to the BeWo cells was determined using various inhibitors. Cytoadhesion assays were carried out as described recently [15]. Binding specificity was determined either by pre-treating the BeWo cells with 0.5 units/ml of chondroitinase ABC (Fluka) or 25 units/ml *Streptomyces hyaluronlyticus* hyaluronidase (Calbiochem) or by pre-incubating the cells with 5 µg/ml anti-CD36 monoclonal antibody FA6/152 (Beckman Coulter) or 5 µg/ml anti-ICAM-1 monoclonal antibody 84H10 (Beckman Coulter) for 1 h at 37°C. Whereas enzyme treated cells were washed prior to IE adhesion, antibodies remained present during adhesion assays. IE were pre-incubated with the soluble inhibitor protein A from *Staphylococcus aureus* (200 µg/ml) or with 200 µg/ml BSA for 20 min at RT. Cytoadhesion assays were carried out in panning buffer pH 7.2. Pictures of cells were taken with a Nikon camera after fixing cells with 2% glutaraldehyde in PBS.

### Flow adhesion assays

Adhesion assays to normal human placenta cryosections under flow conditions at a shear stress of 0.05 Pa were performed as described [43].

### Flow cytometry

Cultures with 3–5% parasitemia synchronous at the mid/late trophozoite stage were washed three times in PBS/0.2% BSA and resuspended in PBS/0.2% BSA to 1×10^7^ cells/ml. Samples were stained for 30 min at RT with sera pools of malaria-exposed male, primigravidae and multigravidae from Malawi (1∶20 dilution) [44]. Sera were collected from pregnant women near delivery. All women gave written consent for HIV counselling and testing, and for the use of their sera to investigate immunity to malaria. Only HIV uninfected women's sera were used in the present study. Samples from males were collected from fathers of children admitted to the same hospital with malaria, who gave witnessed verbal consent. The use of these sera was approved by the College of Medicine Research Ethics Committee, University of Malawi. After washing three times with PBS/0.2% BSA, IE were incubated with PBS/0.2% BSA containing anti-human IgG Alexa488 (1∶100) (Invitrogen) and 10 µg/ml ethidium bromide for 30 min at RT. IE were washed with PBS/0.2% BSA and fixed in 4% paraformaldehyde/PBS over night at 4°C. Analysis was carried out on a FACScan using CellQuest software (BD Biosciences). Data given are the mean values of the geometric mean fluorescence intensities (FI) (±SD) as determined in two independent experiments.

### RNA extraction and real-time RT-PCR for assaying expression of the var gene family

RNA was extracted from synchronized ring stage parasites ∼10 h post-invasion using TRIZOL LS Reagent (Invitrogen). RNA was treated with recombinant Deoxyribonuclease I (Ambion) to degrade contaminating DNA. cDNA synthesis was performed using Multi-Scribe reverse transcriptase (Applied Biosystems) with random hexamer primers (Applied Biosystems). cDNA was synthesized from 4 µg total RNA and one half of this material was used for each real-time comparison. Real-time reactions were performed at optimized final primer concentration of 0.05 µM–0.5 µM using Biorad ITAQ SYBR green Supermix in 40 µl reactions on an ABI Prism 7000. Each *var* gene comparison was performed in triplicate using cDNA from at least two different RNA preparations. Real-time primers and optimized primer concentrations are listed in Table S1. PCR cycling conditions were 50°C for 1 min, 95°C for 3 min followed by 40 cycles of 95°C for 15 s, 52°C for 15 s and 60°C for 1 min. To validate the IT4 *var* primer pairs, they were first tested by real-time PCR on genomic DNA and the median cycle threshold (C_T_) value was calculated. Using published criterion [18], all primer pairs were optimized until individual C_T_ values were within 1.5 cycles of the median C_T_ value. The specificity of each primer pair was confirmed by direct sequencing of PCR products amplified from genomic DNA.

For *var* transcriptional analyses, *var* gene normalization was done by comparison to adenylosuccinate lyase (PFB0295w). Adenylosuccinate lyase was chosen as a control because it displays relatively uniform transcription throughout the bloodstage parasite development [45]. Two other control genes, glutaminyl-tRNA synthetase (PF13_0170) and arginyl-tRNA synthetase (PFL0900c) that have been employed in previous *var* transcripition analysis [46] were included in some reactions and gave similar results to adenylosucciante lyase. The IT4 *var* primer pairs and control primers were first shown to have similar amplification efficiencies by performing amplifications using different concentrations of genomic DNA template. After optimizing primers efficiencies, residual primer bias was corrected by calculating the average difference in C_T_ values between each optimized IT4 *var* primer pair and adenylosuccinate lyase using genomic DNA as template, as described previously [46]. Following *var* transcriptional analyses, the Δ C_T_ for each individual primer pair was determined by correcting for primer bias using the above calculation, and then subtracting the individual C_T_ value from the C_T_ value of the control adenylosuccinate lyase. Δ C_T_s were converted to relative copy numbers with the formula 2^ΔCT^ (User bulletin 2, Applied Biosystems, http:www.appliedbiosystems.com).

### Northern blot Analysis

Total RNA was prepared from synchronized parasite cultures approximately 10 h and 30 h post-invasion, respectively. RNA preparation, electrophoresis, membrane transfer and hybridization were carried out as previously described [47]. The membrane was hybridized at high stringency conditions at 60°C overnight and washed twice with 0.5× SSC, 0.1% SDS at 60°C for 30 min. The probe for FCR3 *var2csa* DBL1-X was generated by PCR amplification from FCR3 genomic DNA using the primers 5′-tccccgcggcattcagattctggaaagtatgatcc-3′ and 5′-ggactagtttcaagagacgaataattagcttcaag-3′ and radiolabelling as previously described [19].

## Supporting Information

Table S1 (0.09 MB DOC)

Figure S1 Transcriptional analysis of *var* genes Northern blot analysis of total RNA isolated from ring (R) and trophozoite stage parasites (T) FCR3-CSA, FCR3-CD36, 1F1-CD36 and 1F1-BeWo. The membrane was hybridized with probes specific for *var2csa* DBL1, *var6* DBL3γ, *var7*, *var34*, *var47* and semi-conserved *varT11.1* exon II. The Northern blot data confirms the quantitative real-time PCR data that several different full-length *var* genes are transcribed in the multi-phenotypic parasite population 1F1-BeWo. The probes were generated by PCR amplification from FCR3 genomic DNA and radiolabelling as previously described [19]. For the *var* probes following primers were used: *var6*: gaagacgaaaattatgcgtaagtag and caagcgttagcacaactagtcttaccg, *var7*: ggacattgttggaaagacagtgc and cattctgcccattcagtcatcc, *var34*: caaaccaaaaccagatggaggtc and cgtgtattcgccgttgtccttg, *var47*: aaccacaagatagtgctggcggac and cttcaaggtaacggaaataagggg.
(1.55 MB DOC)
