# Supplementary material for: Disruption of Var2csa Gene Impairs Placental Malaria Associated Adhesion Phenotype
Source: PLoS One. 2007 Sep 19;2(9):e910. doi: 10.1371/journal.pone.0000910 (PMC1975670; doi:10.1371/journal.pone.0000910)
Supplement: Table S1 — (0.09 MB DOC) [file pone.0000910.s001.doc]

Table S1 Primer list

| **Primer Name** | **Forward Primer** | **Reverse Primer** | **[Final Reaction]** |
| --- | --- | --- | --- |
| Var1 | GATCCGCCAGCAAAAGAAG | CCCCCTTTATATTTTTGTCTGC | 50nM |
| Var2 | GGATGTTGGAGAGGAGTGTTAA | GTCCACTTGATTTAGCGGC | 100nM |
| Var3 | AGCCATGACTTGTGTTGCAC | TCCACACTTTGGATGTGTCAA | 150nM |
| Var4/VAR2CSA 5' | TTGGATAGTGTGTCAGACGAAG | TTTCTTTGCAGTTCGTACCC | 125nM |
| Var4/VAR2CSA 3' | ACGAACGAAAATTGCAGGTT | CGTCTATCACAGAAATTTTCGC | 100nM |
| Var5 | AAAATGGTGATGGAGCCAAG | CGTTGTTTTCGCCACTGAA | 125nM |
| Var6 | CTTTCGTCACCAACCATATGA | AAAGAACCCCCTTCACCAC | 125nM |
| Var7 | TCTCTAGGCCGACGTTCTAA | TCCCTGTGGTCTCTTGTTGT | 80nM |
| Var8 | TGGTGTGCTTGTTCCTCCT | GCTTCAGTGTAGGCAGATTGA | 100nM |
| Var9/R29 | TGGACACATTAAAGGAATGTCA | TGCTTATCCTCATCGTGGT | 100nM |
| Var10 | AAAATGGTCGTAGCGGAAGTG | TGTACATGGTCCACCTGCAGT | 100nM |
| Var11 | CATGGAATGATCTGTGCCC | TTGGAGGTGCCTTTGTCAC | 500nM |
| Var12/ITGvar | CAACCACATTGAAGACGAGGA | CCTCTGGTTGTGGTTGTGTTT | 250nM |
| Var13 | GTAAACATCAGGCGTGTAAGG | TGTTCCTCTCCGCTGAAGA | 500nM |
| Var14/A4var | CAAGATGGAAGCGGTAAAG | CATGCATTATCCCAAAGAT | 125nM |
| Var15/FCR3var2 | TTCTGACTCCTTCGACGACCA | TCCTCTTTCTCCTCGTCTTCG | 150nM |
| Var16/ICAM1 | ATGGTAGACAAGCTGTTCGTTT | AGCACAGGCTCCTACTGAATT | 150nM |
| Var17 | AAACTATACCCCGTGCTTGC | GGCCTTCAACGCATTTCTAT | 200nM |
| Var18 | TTACAGACGCGATTGAAAGAA | CATTGATATATTTCCCGGCA | 100nM |
| Var19 | GCCGAATTTTTGAGATCGA | TCGCCTCTACGTCTGACACTA | 80nM |
| Var20/FCR3var3 | ACCTAGCAGAGGCACCATCAA | TCCTCTTCTGGCTTAGAACGG | 150nM |
| Var21/FCR_S1.2 | AAAGCTTGGCGTAAAAGCAA | GGTTTTGGTCTTCTTCATCGG | 250nM |
| Var22 | AAAGAATGCAAACCATACGC | CTTTCCACGTTATAGTTTCCG | 100nM |
| Var23 | GTCAAAAATGCTGAGGCTAAA | GCATGGATCATTGGTGTCA | 100nM |
| Var24 | GAAAACCCAGCACAGTGTGA | GTGCCATCTGTTTCGTCCT | 100nM |
| Var25 | GCAGAGGTAACAGATCCGTCA | TGGTTGAACAGGCTGCTTG | 100nM |
| Var26 | CTCTGACGCTGCAAGCTTT | TGAGAGCATGGATCACAATG | 500nM |
| Var27 | CAATAACGACAACCCTGGCA | TGGTGTCTTCGTCGGTTTTT | 80nM |
| Var28 | TGATGTCCCAGTTCCTGCT | GGTGTTCGAATGGATTTGG | 100nM |
| Var29 | GCAAATGTGGAGTCCATTTG | CAGTTGGATCTTGTGTTGGTG | 500nM |
| Var30 | TACGAAGAGACAAGGTGTTGG | CGCCATTACCGAGTGTAAGT | 100nM |
| Var31/A4tres | ACTGGTCGTAAAGGTGCACA | CTCCCTTCAAATCACTTCCC | 50nM |
| Var32/CS2 | CAATCAAATGCATATGGTGACC | GCAGCGTCATCTTTTAGTGTTC | 100nM |
| Var33 | AGCACGTTCTGTGGATTTCA | TTCCCAGTCCACACAAAGG | 100nM |
| Var34 | TGGTACTAAGGAGGAGAAGCAG | TTGGAGGCCACTAGCTTTG | 500nM |
| Var35/Var1CSA | AAATGGACCATGTGATGGCA | CATGTGTTCCGGATCCACTT | 125nM |
| Var36 | TCCCAACCTTCTAGTGGAGAA | CCTAATGCACACACCATTCC | 100nM |
| Var39 | TTTACCTGCGTTCCTGAAGTT | GCTGTTGCACTGCTCAATTT | 150nM |
| Var40 | CATACGGAATACTGCGAACC | TGTGGACAATCTGGATGATTT | 100nM |
| Var41 | AACATATGTTTGATAGAATTG | TGGCATCTGTAGGCACGAA | 500nM |
| Var43 | AACACGAAAAGCAAGGATA | TTTTTGTAACTCTTGTTCGG | 100nM |
| Var44 | TGCGAAAGAAGAAGCAGAAG | CACGGCATGTTGTTACCTG | 100nM |
| Var45 | TGCGAATGCAGATGACATAG | CAGTACACCGACCAACAAGAG | 400nM |
| Var46 | TAATGGCACATACGAAACCC | GGAGAATTCCTCCAAGGTTG | 150nM |
| Var47 | CGAAAACCCAACACAGTGTC | AATTTCTGGCGCTTTCACA | 100nM |
| Var51 | GATAGTGATACGGACCGCAA | CCATTCATGTAAGCAACGAAA | 150nM |
| Var54 | CGAATGTAAAGCAGTCACTCC | TGGTGCAGATGGAAGTTTCT | 50nM |
| Var58 | CATGACGAGGAGGATTATCAA | TGCCTTCACTTTCGATCTTT | 50nM |
| Var60 | AGCATTCGATGAAAAACCAA | CGTAATTTGAAGGCAGCTTT | 100nM |
| Var64 | CGAAGAACCTCGCAATCAA | CCCTTCTGCCACATCTTTCAC | 100nM |
| AFBR6 | GTGTGGAAAGCCATCACGT | TGTGATTAGCTTCGGCTCC | 125nM |
| AFBR7 | GCTCGCTACAAAGATGGAAA | GCCTTACATGTGATGGCCT | 100nM |
| AFBR20 | GGATAAGGAGGAAGCAAAAAA | CCACTCTTAACGTCACACGTG | 150nM |
| AFBR38 | GTGGTGCAGGACAAATTGAT | GGAACAGTTCCAATAGCGC | 80nM |
| FCR3var13 | ACATGACAAATTGGATGATAGC | TACTGTTTCTCGATTCGCC | 100nM |
| FCR3var57 | GCTCTAAAAACACGCTACGG | TGTTAATGCTTCCCACACTGT | 200nM |
| FCR3varc28 | CACGCTACCAAGATGATGGA | CACATGTTAATGCTTCCCAGA | 175nM |
| Adenylosuccinate lyase | CTCATCATTTGCGTATTCCCT | GCTAATGTGCACCAATTTTGA | 100nM |
| Seryl-tRNA synthetase | AAGTAGCAGGTCATCGTGGTT | TTCGGCACATTCTTCCATAA | 150nM |
| Arginyl-tRNA synthetase | AAGAGATGCATGTTGGTC | GTACCCCAATCACCTACA | 100nM |
| Glutaminyl-tRNA synthetase | GGCACTTCAAGGGTACCT | TAATATAGCCTCACAAGC | 400nM |
