# Supplementary figures and images for: Disruption of Var2csa Gene Impairs Placental Malaria Associated Adhesion Phenotype
Source: PLoS One. 2007 Sep 19;2(9):e910. doi: 10.1371/journal.pone.0000910 (PMC1975670; doi:10.1371/journal.pone.0000910)

**Figure S1**


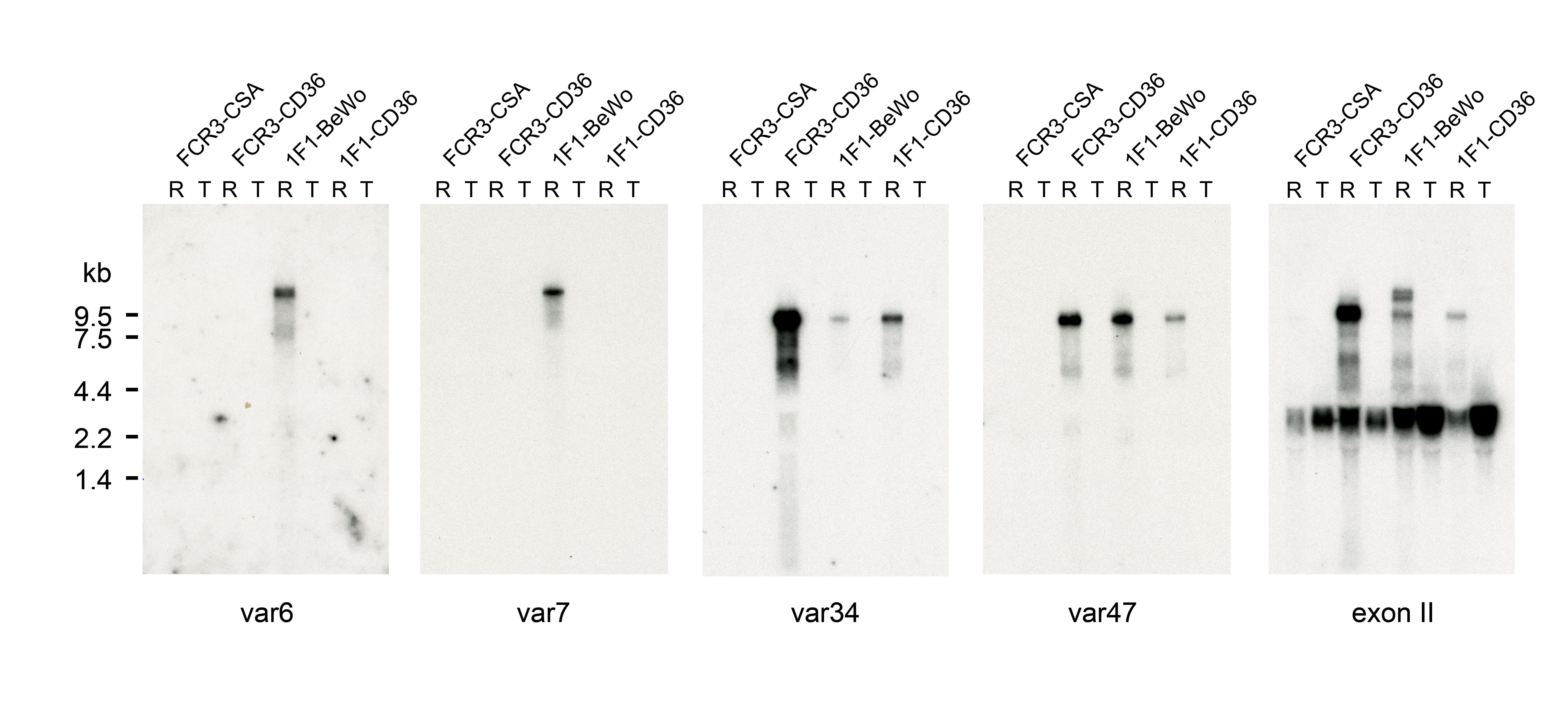

Supplement: Figure S1 — Transcriptional analysis of var genes Northern blot analysis of total RNA isolated from ring (R) and trophozoite stage parasites (T) FCR3-CSA, FCR3-CD36, 1F1-CD36 and 1F1-BeWo. The membrane was hybridized with probes specific for var2csa DBL1, var6 DBL3γ, var7, var34, var47 and semi-conserved varT11.1 exon II. The Northern blot data confirms the quantitative real-time PCR data that several different full-length var genes are transcribed in the multi-phenotypic parasite population 1F1-BeWo. The probes were generated by PCR amplification from FCR3 genomic DNA and radiolabelling as previously described [19]. For the var probes following primers were used: var6: gaagacgaaaattatgcgtaagtag and caagcgttagcacaactagtcttaccg, var7: ggacattgttggaaagacagtgc and cattctgcccattcagtcatcc, var34: caaaccaaaaccagatggaggtc and cgtgtattcgccgttgtccttg, var47: aaccacaagatagtgctggcggac and cttcaaggtaacggaaataagggg. (1.55 MB DOC) [file pone.0000910.s002.doc]
